# Geographic mobility and treatment outcomes among people in care for tuberculosis in the Lake Victoria region of East Africa: A multi-site prospective cohort study

**DOI:** 10.1371/journal.pgph.0001992

**Published:** 2023-06-05

**Authors:** Grace E. Mulholland, Michael E. Herce, Ubaldo M. Bahemuka, Zachary A. Kwena, Kidola Jeremiah, Brenda A. Okech, Elizabeth Bukusi, Elialilia S. Okello, Gertrude Nanyonjo, Ali Ssetaala, Janet Seeley, Michael Emch, Audrey Pettifor, Sharon S. Weir, Jessie K. Edwards

**Affiliations:** 1 Department of Epidemiology, University of North Carolina at Chapel Hill, Chapel Hill, North Carolina, United States of America; 2 Institute for Global Health and Infectious Diseases, School of Medicine, University of North Carolina at Chapel Hill, Chapel Hill, North Carolina, United States of America; 3 Medical Research Council/Uganda Virus Research Institute & London School of Hygiene and Tropical Medicine, Uganda Research Unit, Entebbe, Uganda; 4 Kenya Medical Research Institute, Nairobi, Kenya; 5 Mwanza Intervention Trials Unit, Mwanza Research Centre, National Institute for Medical Research, Mwanza, Tanzania; 6 UVRI-IAVI HIV Vaccine Program Limited, Entebbe, Uganda; 7 Global Health and Development Department, London School of Hygiene and Tropical Medicine, London, United Kingdom; 8 Department of Geography, University of North Carolina at Chapel Hill, Chapel Hill, North Carolina, United States of America; Africa Health Research Institute, SOUTH AFRICA

## Abstract

Geographic mobility may disrupt continuity of care and contribute to poor clinical outcomes among people receiving treatment for tuberculosis (TB). This may occur especially where health services are not well coordinated across international borders, particularly in lower and middle income country settings. In this work, we describe mobility and the relationship between mobility and unfavorable TB treatment outcomes (i.e., death, loss to follow-up, or treatment failure) among a cohort of adults who initiated TB treatment at one of 12 health facilities near Lake Victoria. We abstracted data from health facility records for all 776 adults initiating TB treatment during a 6-month period at the selected facilities in Kenya, Tanzania, and Uganda. We interviewed 301 cohort members to assess overnight travel outside one’s residential district/sub-county. In our analyses, we estimated the proportion of cohort members traveling in 2 and 6 months following initiation of TB treatment, explored correlates of mobility, and examined the association between mobility and an unfavorable TB treatment outcome. We estimated that 40.7% (95% CI: 33.3%, 49.6%) of people on treatment for TB traveled overnight at least once in the 6 months following treatment initiation. Mobility was more common among people who worked in the fishing industry and among those with extra-pulmonary TB. Mobility was not strongly associated with other characteristics examined, however, suggesting that efforts to improve TB care for mobile populations should be broad ranging. We found that in this cohort, people who were mobile were not at increased risk of an unfavorable TB treatment outcome. Findings from this study can help inform development and implementation of mobility-competent health services for people with TB in East Africa.

## Introduction

In 2019, an estimated 365,000 people fell ill with tuberculosis (TB) across Kenya, Tanzania, and Uganda [1]. Despite the availability of effective treatment, TB remains among the world’s most deadly infectious diseases and a leading cause of death in low and middle income countries [2]. Among people who initiate TB treatment, treatment continuity is important for reducing the risks of unfavorable TB treatment outcomes (i.e., death, loss to follow-up, or treatment failure) and drug resistance [3–5], yet adherence to the full course of TB treatment can be challenging. The recommended standard of care for people newly initiating treatment for drug-susceptible TB is a 6-month regimen involving daily dosing of multiple anti-TB drugs [6–10]. Treatment durations are even longer for people with drug-resistant TB [11].

Overnight travel can disrupt daily routines and bring a person far from the area where they are enrolled in TB treatment. It can interfere with adherence to the prescribed daily medication regimen and missed clinic visits. At routine clinic visits, treatment progress is monitored, potential side effects are evaluated, and people receive their next supply of anti-TB drugs. Missed visits can therefore causes lapses in medication supply and prevent timely intervention when treatment is unsuccessful or a person experiences severe adverse events. Health care workers have reported difficulty in following up with mobile populations after initiation of TB treatment [12], and studies in the region have reported travel as a common reason for treatment interruption [13–16]. Through these mechanisms, mobile populations may be at greater risk of unfavorable TB treatment outcomes.

TB treatment among mobile populations may be of particular concern in settings such as the Lake Victoria region of East Africa, where movement of people across national borders is common but there is little cross-border collaboration among health facilities. Many mobility-related barriers to care have been described in the HIV literature [17–19]. In the Lake Victoria region, HIV-focused studies have discussed the movements of people around the lake [19–26], and an association between mobility and missed HIV clinic visits has been reported [22]. Among people with TB, much of the existing mobility-related research has focused on migrants’ risk of acquiring or transmitting TB [27, 28]. Little is known about mobility among people on TB treatment or the impact of mobility on treatment outcomes.

In this study, we describe geographic mobility among people who initiated TB treatment at 12 public sector health facilities in the Lake Victoria region of Kenya, Tanzania, and Uganda. We also estimate the association between geographic mobility while on TB treatment and clinical and demographic characteristics. Finally, we estimate the association between mobility and unfavorable TB treatment outcomes encompassing treatment failure, death, or loss to follow-up.

## Methods

### Study population

**Fig 1:**
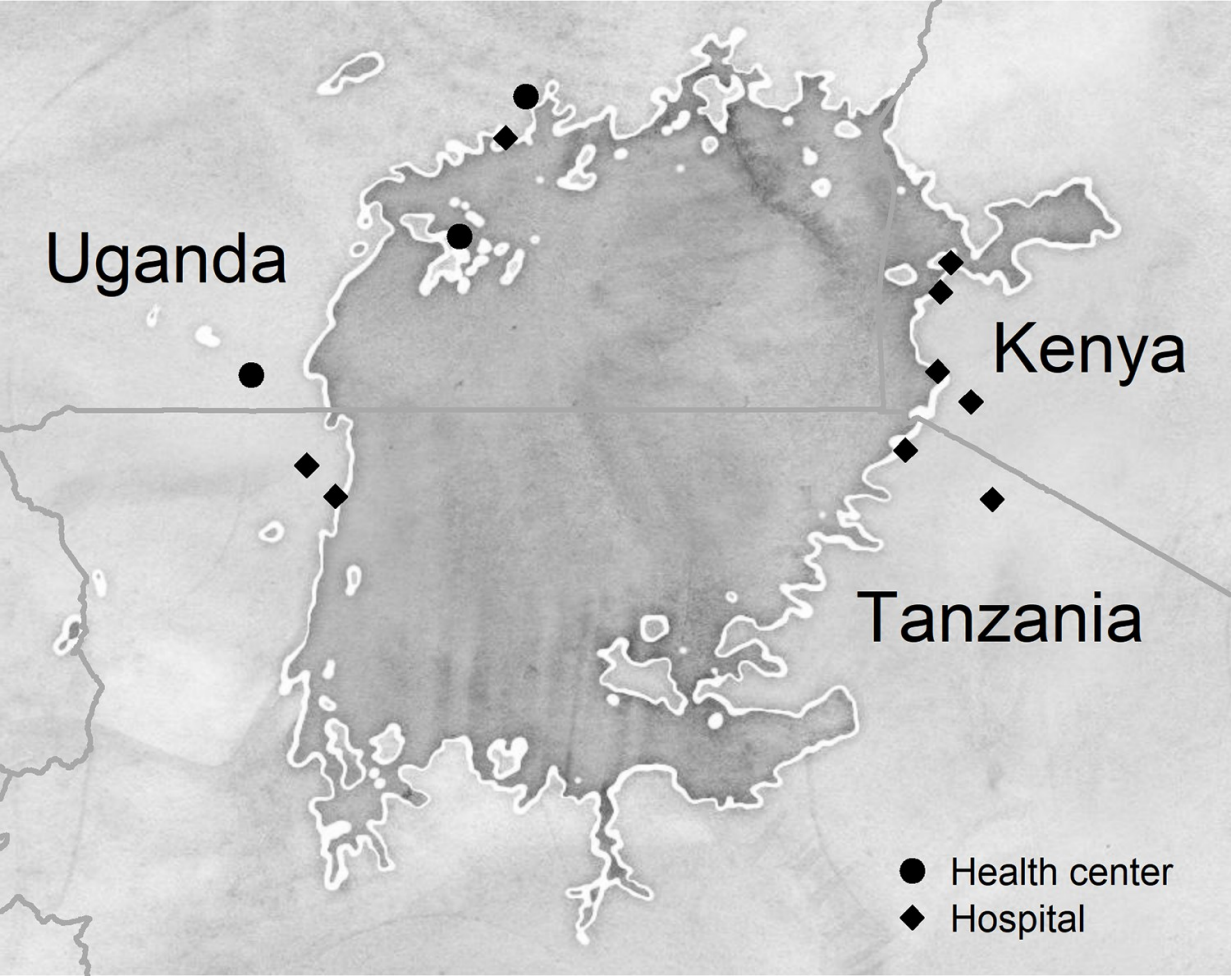
Health facilities included in this study. These facilities were selected for enumeration of a cohort of people on TB treatment in the 2019 East Africa TB/HIV and Mobility Study. Map data are from OpenStreetMap (openstreetmap.org/copyright); map tiles by Stamen Design (http://maps.stamen.com/#watercolor/8/-1.300/32.900).

The study population consisted of all adults (i.e., those 18 and older), irrespective of sex, who initiated TB treatment at one of 12 public sector health facilities located near the shores of Lake Victoria in the 6 months preceding baseline data collection. Facilities were selected following scoping visits to health facilities in the region using the following criteria: high volume of people on TB treatment, availability of or linkage to Xpert MTB/RIF diagnostic testing, capacity to initiate anti-TB therapy on site and treat people with drug-susceptible and drug-resistant TB, maintenance of person-level TB treatment records, and willingness to participate and facilitate access to treatment records in accordance with study procedures. Data were collected at health centers and hospitals in the districts of Kyotera, Kalangala, and Wakiso in Uganda; in Suba, Mbita, Karungu, and Macalder sub-counties in Kenya; and in the districts of Shirati, Tarime, and Bukoba in Tanzania (Fig 1).

### Data collection

#### Overview of procedures

Partner research institutions in Kenya, Tanzania, and Uganda recruited one local data collection team per country. Each team was composed of men and women skilled in research methods and in the languages used by people attending the selected health facilities. The data collection teams were trained jointly in the study procedures and instruments. When collecting data, teams used country-specific tablet-based forms programmed to match the fields in each country’s TB treatment registers and cards. Survey instruments were also translated into the local languages spoken by people visiting the health facilities.

The study team reviewed TB treatment records and applied study eligibility criteria to enumerate the study population (the “cohort”) and abstract basic clinical and demographic data. Then, they recruited a subset of cohort members (a “subcohort”) for a survey assessing additional clinical and demographic characteristics and geographic mobility in the preceding 6 months. At study closure, the data collectors returned to the health facility and abstracted available TB treatment outcome data for the cohort.

#### Enumeration of the cohort and abstraction of medical records for the cohort

Between May and September 2019, the study team visited the health facilities and reviewed TB treatment registers to construct a cohort of people recently enrolled in TB treatment. All people who enrolled in treatment at each facility over the 6-month period were included in the cohort (n = 776). At the start of the study, the study team abstracted key clinical and demographic variables for all cohort members from clinic TB registers and, where available, TB treatment cards. Abstracted variables included health facility and country of treatment initiation, date of treatment initiation, age at treatment initiation, sex, current HIV status, and patient type (new, relapse, or treatment after failure or loss to follow-up), TB site (pulmonary or extra-pulmonary), diagnostic testing results, and area of residence.

#### Recruitment of a subcohort and subcohort survey

Due to budget and time constraints, we conducted a survey to collect demographic, clinical, and mobility among a subset of cohort members rather than the full cohort. Following initial records abstraction, the study team recruited a subcohort of 301 (38.8%) of the 776 cohort members to participate in a survey. Briefly, as cohort members visited a participating health facility for routine care, members were recruited to participate in the subcohort survey according to prespecified recruitment targets. The targets ensured that the subcohort represented the proportionate distribution of cohort members across health facilities, and that at each facility, the subcohort included an approximately equal number of subcohort members with and without HIV. Further details of the subcohort recruitment process are provided in S1 Text.

The study team conducted face-to-face interviews among cohort members recruited for the subcohort survey, asking questions related to socio-demographics, TB care experiences, and other characteristics not captured in routine health records. Subcohort members were also asked to list all overnight trips they took outside their geographic area of residence in the prior 6 months. For participants residing in Uganda and Tanzania, the area of residence was defined at the district level. Kenya does not currently use districts as subdivisions; the sub-county was considered the most analogous administrative area and was used to define area of residence among participants residing in Kenya.

Interviewers used printed calendars to guide each participant in generating a complete calendar of overnight travel outside the residential area in the 6 months preceding the interview date. Interviewers then collected and entered details for each trip, including the destination and reason for travel.

#### TB treatment outcome ascertainment for the cohort

Between 3 and 6 months after enumerating the cohort, the data collection team returned to the health facilities and consulted TB registers to abstract treatment outcomes for all cohort members. At this time, fifteen cohort members (1.9%) had surpassed the typical duration of TB treatment (6 months) with no outcome recorded. Following abstraction of treatment outcomes, data collectors performed a phone-based tracing study to ascertain/confirm treatment outcomes among subcohort members who initiated treatment approximately 6 months or more before study closure but had no outcome recorded or had recorded outcomes of “lost to follow-up” or “not evaluated.” For those who were successfully traced, we updated their TB treatment outcomes to the outcomes they reported in the tracing study.

### Ethical considerations

The study protocol was approved by the institutional review boards of the University of North Carolina at Chapel Hill, the Kenya Medical Research Institute, the Uganda Virus Research Institute Research Ethics Committee, the Uganda National Council for Science and Technology, the London School of Hygiene and Tropical Medicine Research Committee, and the Medical Research Coordinating Committee of the National Institute for Medical Research in Tanzania. When recruiting participants for the subcohort survey, the study team provided details about the study and obtained written informed consent before conducting the interviews. Participants received compensation for transportation and time spent in the interview. Data were entered into encrypted tablet-based forms using Open Data Kit software and were aggregated on a secure server.

### Statistical methods

#### Geographic mobility measures

We used 30-day periods to represent each “month” of follow-up since initiation of TB treatment. The number of reported “months” of mobility data while on treatment varied across subcohort members. This is because subcohort members were recruited upon visiting the health facility during the subcohort recruitment stage of data collection (as opposed to being recruited at a specific point in TB treatment). For example, a subcohort member who initiated TB treatment one month before participating in the survey could contribute one month of data describing their mobility while on TB treatment. A subcohort member who initiated TB treatment five months before they participated in the survey could contribute five months of data describing their mobility while on treatment. Mobility data were considered missing for months in which only part of a 30-day period was described.

#### Imputation of missing data and coding of mobility analysis variables

We used a multiple imputation approach with fully conditional specification (FCS) [29, 30], also known as multivariate imputation of chained equations (MICE), to impute the following sets of binary indicator variables related to mobility in the months that followed initiation of TB treatment:

1. Whether, in each month 1 through 6, the cohort member initiated an overnight trip outside their area of residence;
2. Whether, in each month 1 through 6, the cohort member traveled overnight outside their area of residence for more than 7 total nights (even if on separate trips); and
3. Whether, in months 1 or 2, the cohort member initiated an overnight trip outside their area of residence that lasted longer than 14 consecutive days. We considered a period of 14 days because such a trip would exceed the typical 1- or 2-week interval between anti-TB drug refills during the intensive phase of TB treatment [31–33].

We fit imputation models that included variables suspected to be associated with mobility or missing mobility data and the mobility variables. We included clinical, demographic, socioeconomic, and mobility variables, an outcome indicator, and the cumulative baseline hazard [34]. Missing values were imputed for the mobility variables and other model covariates. The imputation models were congenial with our analysis models [35]. Further details of the imputation model and procedure are provided in S2 Text. We produced 200 “complete” data sets, and when analyzing the data, we used Rubin’s rules [36] to allow uncertainty in the imputation process to propagate through to final confidence intervals around our estimates.

#### Estimating probability and correlates of geographic mobility

For our main analyses, we coded a mobility variable that indicated whether a person took any overnight trips outside their area of residence within the first 2 or 6 months following initiation of TB treatment. These periods were selected to align with the typical duration of the intensive phase of treatment (2 months) and the typical total duration of TB treatment (6 months).

To estimate the overall probability of travel in 2 months or 6 months following initiation of TB treatment, we fit an intercept-only log-binomial model in each imputation. For each covariate, we then fit separate log-binomial models to estimate the probability of travel in each period (2 and 6 months) in each level of the covariate. We compared travel probabilities across levels of each covariate to estimate the association between the covariate and mobility in each period.

#### Estimating the association between geographic mobility and risk of TB treatment outcomes

We coded “unfavorable TB treatment outcome” as a composite outcome that included death, loss to follow-up, and treatment failure. We followed cohort members from their date of TB treatment initiation until the first date of unfavorable TB treatment outcome, date of transfer or outcomes listed as “unknown” or “not evaluated,” 200 days following treatment initiation, or facility-specific date of study closure (i.e., date of outcome data collection). Missing death dates (n = 6) were imputed as the midpoint between the person’s last recorded contact with the health facility and the date of study closure, and missing dates for loss to follow-up (n = 14) were imputed as the date 60 days beyond the person’s last recorded contact with the health facility, to approximate a treatment interruption of 2 months [37]. The eligibility criteria for cohort members allowed for the date of TB treatment initiation to vary by up to 6 months within a single facility, and data collection did not open and close simultaneously across all 12 sites. As a result, the duration of TB treatment experienced by the time of study closure varied across cohort members. Cohort members with no recorded treatment outcome and with less than 6 months between initiation of treatment and outcome ascertainment (n = 122) were censored at study closed and assigned the outcome “Presumed still on treatment: On treatment for less than 6 months and no outcome recorded.”

We used the Nelson-Aalen estimator [38, 39] to compute the risk, or cumulative probability, of experiencing an unfavorable TB treatment outcome under each of the following mobility patterns:

1. Mobility as observed: The mobility patterns observed or imputed (based on observed data) in the cohort (i.e., the “natural course”)
2. Limited travel: No travel during the first 2 months of TB treatment; mobility patterns as observed thereafter
3. No travel: No travel during the first 6 months of TB treatment

We estimated risk by 60 and by 180 days since treatment initiation. To estimate risks under the “limited travel” pattern, we censored participants from the risk set at the end of the month in which they took their first trip after initiating TB treatment if that first trip occurred within 2 months of starting treatment. Otherwise, participants were followed until their observed outcome or censoring time. To estimate risks under “no travel,” we censored participants at the end of the month in which they took their first trip following initiation of TB treatment. We accounted for informative censoring induced by deviations from each prescribed mobility pattern using inverse probability of censoring weights [40]. The censoring approach and models used to compute censoring weights are further described in S3 Text. We used the delta method to estimate standard errors of the risk differences [41].

Analyses were performed in SAS 9.4 [42] and R 4.0.5 [43]. We used map data from OpenStreetMap, licensed under the Open Data Commons Open Database License (ODbL) by the OpenStreetMap Foundation (OSMF) [44]. Map tiles are by Stamen Design, licensed under CC BY 3.0 [45].

## Results

Table 1 presents the characteristics of the cohort and subcohort. In the cohort, 13.5% of members were recruited from Kenya, 44.0% were recruited from Tanzania, and 42.5% were recruited from Uganda. Women comprised 40.9% of the cohort. The median age of cohort members was 39 (interquartile range: 29–50) years. Most cohort members (86.1%) were diagnosed with pulmonary TB, and over 90% were new TB cases. Approximately half of the cohort members (49.7%) had HIV-associated TB. TB had been bacteriologically confirmed (based on sputum smear microscopy, Xpert, and culture captured in the treatment register or card) for just under half of the cohort (47.4%); there was a high degree of missingness in the records, with, for example, 28.5% of cohort members having no Xpert testing recorded at the time of initial records abstraction. Drug-resistant TB was documented for only a small fraction of cohort members (2.6%). Among cohort members with a documented residence, more than 80% resided in the same district (in Kenya, sub-county) as the health facility where they initiated TB treatment. The distribution of demographic and clinical features in the subcohort (n = 301, 38.8% of the cohort) closely resembled that observed in the cohort.

**Table 1:** Characteristics of people initiating TB treatment over a 6-month period at 12 health facilities near Lake Victoria.

Characteristics ascertained in initial health facility records abstraction ^a^	Subcohort (n = 301)		Full cohort (n = 775)	
	n	%	n	%
Female	131	43.5	317	40.9
Age group				
18 to 24 years	36	12.0	92	11.9
25 to 34 years	73	24.3	199	25.7
35 to 49 years	124	41.2	276	35.6
50 years or older	68	22.6	208	26.8
Country of treatment initiation				
Kenya	49	16.3	105	13.5
Tanzania	125	41.5	341	44.0
Uganda	127	42.2	329	42.5
TB site				
Pulmonary	262	87.9	659	86.1
Extrapulmonary	36	12.1	106	13.9
Missing	3		10	
Patient type				
New	265	89.5	706	91.9
Relapse	27	9.1	56	7.3
Treatment after failure	1	0.3	1	0.1
Treatment after loss to follow-up	3	1.0	5	0.7
Other (unspecified)/Missing	5	1.7	7	
HIV-associated TB	157	52.2	386	49.8
TB bacteriologically confirmed by:	160	53.2	367	47.4
GeneXpert	141	46.8	321	41.4
Culture	25	8.3	50	6.5
Smear	16	5.3	36	4.6
Unspecified method	5	1.7	11	1.4
Drug-resistant TB ^b^	16	5.3	20	2.6
Resided in area ^c^ where health facility is located				
Yes	177	86.3	400	80.0
No	28	13.7	100	20.0
Missing	96		275	
Participated in subcohort survey	301	100.0	301	38.8
**Additional characteristics assessed in subcohort survey** ^**d**^				
Marital status				
Married or cohabitating with sexual partner	152	50.5		
Separated/Divorced	71	23.6		
Single (never married)	42	14.0		
Widowed	36	12.0		
Educational attainment				
Less than primary school (including no school)	118	39.2		
Primary school	140	46.5		
Form 6	12	4.0		
College (vocational, tertiary or non-tertiary) or university	31	10.3		
Employment status				
Formally employed	50	16.6		
Informally employed	157	52.2		
Not employed, seeking work	44	14.6		
Not employed, not seeking work	50	16.6		
Worked in the fishing industry in the past 12 months				
Yes	47	15.6		
No	247	84.4		
Missing	7			
Worked in a mine in the past 12 months				
Yes	32	10.7		
No	257	89.3		
Missing	12			
Any household members went to bed hungry in the past 4 weeks				
Yes	85	28.9		
No	209	71.1		
Missing	7			
Time from TB symptom onset to presenting to clinic				
No more than 6 weeks	134	45.9		
More than 6 weeks	158	54.1		
N/A: Reported having no symptoms prior to care	9			

Data are from the 2019 East Africa TB/HIV and Mobility Study.

^a^ Variables recorded in TB registers were abstracted for the full analytic cohort.

^b^ Includes additional diagnoses noted by time of study closure.

^c^ District in Uganda and Tanzania; sub-county in Kenya.

^d^ These variables were not recoded in TB registers and were assessed only among a subset of the cohort (a “subcohort”).

Across the 301 subcohort members, mobility data were reported for 980 complete person-months, with each participant contributing 0 to 6 (mean = 3.3, SD = 1.6) complete months of mobility data. Participant-reported mobility data are described in S1 Table. Briefly, an overnight trip away from a cohort member’s residential area was initiated in 7.2% of described months. Disaggregated by sex, overnight trips were initiated in 8.5% and 5.5% of described months among male and female cohort members, respectively. Overnight trips were initiated in 7.2% and 8.1% of described months among cohort members with HIV-associated TB and those without HIV, respectively. Sex- and HIV status-disaggregated patterns were similar when considering any nights spent away from the residential area during a month, with nights away reported for 10.3%, 7.2%, 7.9%, and 10.2% of person-months described by male participants, female participants, participants with HIV-associated TB, and participants without HIV, respectively. In months during which people spent nights away from their residential area, they spent an average of 8.5 (SD = 8.9) nights away. Fifty-seven subcohort members reported a total of 82 trips outside their residential area since initiating TB treatment. The most common reasons for travel during TB treatment were to work or seek work (reported for n = 29, 35.8% of trips), to visit family or friends (for n = 19, 23.5% of trips), to attend a burial or funeral (for n = 14, 17.3% of trips), and holiday/vacation (for n = 6, 7.4% of trips). Additional reasons are presented in S1 Fig.

**Fig 2:**
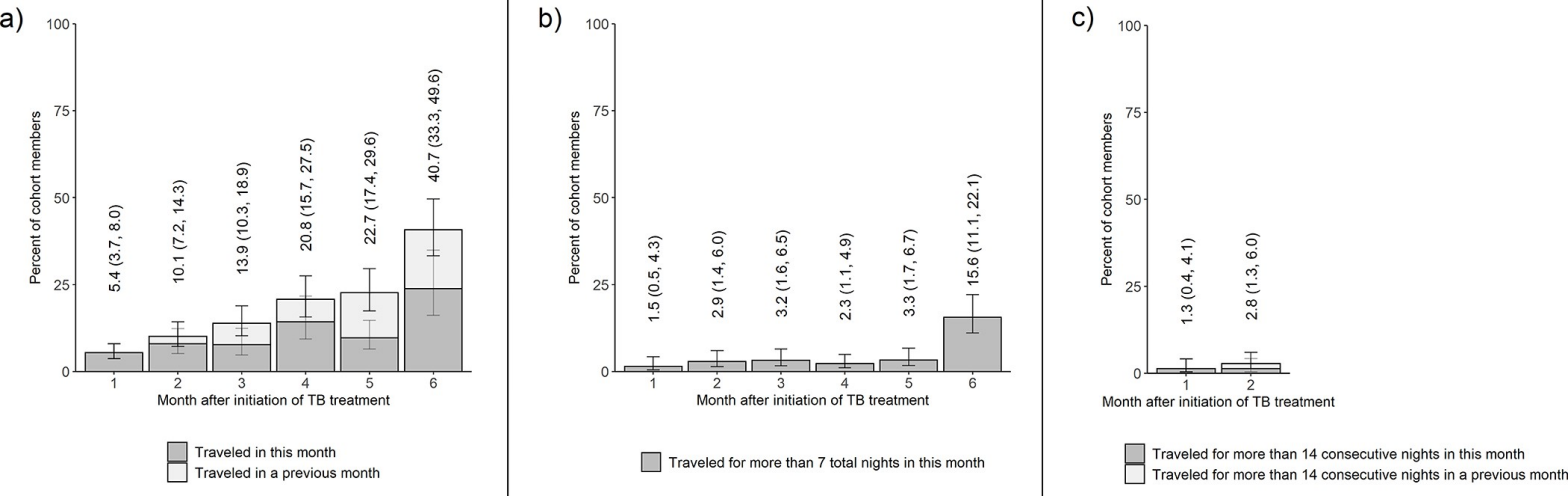
Estimates of mobility measures among cohort members in the months following TB treatment initiation. Results are estimates for overnight travel outside one’s area of residence. Figures show the percentages of cohort members estimated to have a) traveled in or before the month (value labels show the cumulative percentages traveling by each month), b) traveled for more than 7 total nights in the month, and c) initiated a trip of 14 or more consecutive nights in or before the month (value labels show cumulative percentages). 95% confidence intervals are shown parenthetically. Data are from the 2019 East Africa TB/HIV and Mobility Study.

Following imputation of missing data, we estimated that 40.7% (95% CI: 33.3%, 49.6%) of people in TB care traveled overnight outside their area of residence in the first 6 months following treatment initiation (Fig 2A). An estimated 10.1% (95% CI: 7.2%, 14.3%) traveled within the first 2 months. In the first month after treatment initiation, we estimated that only 1.5% (95% CI: 0.5%, 4.3%) of people traveled outside their area for more than 7 total nights, and only 2.9% (95% CI: 1.4%, 6.0%) traveled for more than 7 total nights in month 2 (Fig 2B). Long trips during these initial months were also uncommon; by the end of month 2, only 2.8% (95% CI: 1.3%, 6.0%) of people had initiated a trip that lasted longer than 14 consecutive nights (Fig 2C).

The proportion of cohort members traveling was highest in month 6, in which an estimated 23.8% (95% CI: 16.2%, 34.9%) of people on TB treatment traveled overnight outside their area (S2 Table). The proportion traveling did not increase monotonically across months, and the patterns varied slightly across subgroups. The proportion of people traveling in each month, along with results disaggregated by sex, HIV status, TB site, work in the fishing industry, and proximity of residence to health facility are provided in S2 Fig. The proportion of people traveling for more than 7 total nights within a single month increased most from month 5 to 6. In the sixth month since treatment initiation, an estimated 15.6% (95% CI: 11.1%, 22.1%) of cohort members spent more than 7 nights away from their area of residence.

An estimated 11.7% (95% CI: 8.0, 17.0%) of men and 7.8% (95% CI: 4.4%, 13.9%) of women in care traveled within 2 months of initiating treatment (Table 2). Those who had worked in the fishing industry in the past 12 months were 3.28 (95% CI: 1.62, 6.63) times as likely as others to have traveled within 2 months of initiating TB treatment. People with extra-pulmonary TB were 2.99 (95% CI: 1.58, 5.65) times more likely than those with pulmonary TB to travel in the first 2 months. People in care in Kenya were 1.52 times as likely as those in Uganda to travel within 6 months (95% CI: 1.01, 2.28). Associations between other covariates and mobility were smaller and imprecise. Point estimates suggest that older people on TB treatment may be less likely than younger people to travel in the early months of treatment. Those age 50 years or older had a 5.6% (95% CI: 2.3%, 13.3%) probability of travel in the first 2 months, compared to a 13.7% (95% CI: 6.9%, 27.2%) probability of travel among people ages 18 to 24 (probability ratio: 0.41; 95% CI: 0.14, 1.19).

**Table 2:** Percentages of cohort members traveling and associations with participant characteristics.

	Travel ^a^ in the 2 months following TB treatment initiation		Travel in the 6 months following TB treatment initiation	
	Probability (%)	Probability ratio ^b^ (95% CI)	Probability (%)	Probability ratio (95% CI)
Overall	10.1 (7.2, 14.3)	—	40.7 (33.3, 49.6)	—
Sex				
Male	11.7 (8.0, 17.0)	1	46.2 (37.4, 57.1)	1
Female	7.8 (4.4, 13.9)	0.67 (0.36, 1.25)	32.4 (23.3, 45.0)	0.70 (0.50, 0.99)
Age group				
18 to 24 years	13.7 (6.9, 27.2)	1	56.8 (37.2, 86.7)	1
25 to 34 years	11.8 (6.6, 21.3)	0.86 (0.37, 2.02)	32.2 (23.2, 44.8)	0.57 (0.36, 0.91)
35 to 49 years	10.7 (6.8, 16.8)	0.78 (0.35, 1.73)	43.5 (33.2, 57.1)	0.77 (0.50, 1.18)
50 years or older	5.6 (2.3, 13.3)	0.41 (0.14, 1.19)	37.0 (27.1, 50.5)	0.65 (0.39, 1.10)
Country of treatment initiation				
Kenya	14.4 (7.7, 27.1)	1	55.8 (39.0, 79.8)	1
Tanzania	8.0 (4.6, 14.0)	0.56 (0.25, 1.25)	39.4 (30.0, 51.7)	0.71 (0.48, 1.05)
Uganda	10.7 (6.6, 17.4)	0.74 (0.35, 1.58)	36.7 (27.7, 48.7)	0.65 (0.39, 0.99)
TB site				
Pulmonary	7.9 (5.2, 11.9)	1	38.6 (31.1, 47.8)	1
Extrapulmonary	23.6 (13.8, 40.2)	2.99 (1.58, 5.65)	53.3 (38.5, 73.8)	1.38 (0.98, 1.95)
HIV status				
HIV-negative	11.3 (7.5, 16.9)	1	45.9 (36.7, 57.5)	1
HIV-positive	8.9 (5.2, 15.1)	0.79 (0.42, 1.47)	35.2 (26.5, 46.7)	0.77 (0.56, 1.04)
TB bacteriologically confirmed				
Yes	9.6 (6.1, 15.0)	1	41.3 (32.6, 52.5)	1
No	10.5 (6.7, 16.5)	1.10 (0.62, 1.95)	40.0 (31.4, 50.8)	0.97 (0.74, 1.26)
Resided in area ^c^ where health facility is located				
Yes	9.2 (6.3, 13.4)	1	40.0 (32.0, 49.9)	1
No	13.7 (6.3, 30.0)	1.50 (0.63, 3.56)	42.7 (27.2, 66.8)	1.07 (0.65, 1.76)
Time from onset of TB symptoms to first presenting to a health facility				
6 weeks or less	12.5 (7.9, 19.8)	1	42.9 (33.6, 54.7)	1
Greater than 6 weeks	7.9 (4.8, 13.2)	0.63 (0.32, 1.25)	38.6 (29.7, 50.2)	0.90 (0.66, 1.24)
Marital status				
Married or cohabitating with a sexual partner	8.2 (4.6, 14.7)	1	38.1 (29.3, 49.6)	1
Not married or cohabitating with a sexual partner	11.6 (7.7, 17.6)	1.41 (0.70, 2.85)	42.7 (33.8, 54.1)	1.12 (0.83, 1.51)
Highest educational attainment				
Less than primary school (including no school)	16.0 (8.1, 31.7)	0.88 (0.47, 1.68)	37.8 (28.3, 50.5)	0.98 (0.67, 1.43)
Primary school	9.6 (6.1, 15.0)	1	38.5 (29.0, 51.2)	1
Form 6 or higher	7.4 (3.6, 15.4)	0.91 (0.34, 2.46)	51.7 (37.5, 71.2)	1.29 (0.86, 1.94)
Employment status				
Formally employed	16.0 (8.1, 31.6)	1.67 (0.74, 3.79)	39.3 (27.4, 56.6)	1.10 (0.70, 1.72)
Informally employed	9.6 (6.1, 15.0)	1	35.8 (26.8, 48.0)	1
Not employed	7.4 (3.6, 15.4)	0.78 (0.34, 1.80)	48.7 (37.7, 62.9)	1.34 (0.94, 1.91)
Worked in the fishing industry in the past 12 months				
Yes	24.8 (14.9, 41.2)	3.28 (1.62, 6.63)	59.4 (43.3, 81.5)	1.59 (1.12, 2.25)
No	7.6 (4.7, 12.0)	1	37.4 (30.0, 46.7)	1
Worked in a mine in the past 12 months				
Yes	6.5 (1.7, 25.2)	0.62 (0.16, 2.44)	54.7 (39.1, 76.6)	1.40 (0.97, 2.03)
No	10.5 (7.4, 14.8)	1	39.0 (31.4, 48.4)	1
Any household members went to bed hungry in the past 30 days				
Yes	9.8 (6.5, 14.9)	0.92 (0.44, 1.91)	39.6 (31.5, 49.8)	0.91 (0.66, 1.27)
No	10.7 (5.8, 19.7)	1	43.3 (32.4, 57.9)	1

Data are from the 2019 East Africa TB/HIV and Mobility Study.

^a^ Any overnight travel outside the district or sub-county of residence in the stated period.

^b^ Probability ratios compare the probability of travel by demographic and clinical characteristics in the first 2 and 6 months following TB treatment initiation.

^c^ District in Uganda and Tanzania; sub-county in Kenya.

The full distribution of outcomes in the cohort is shown in Table 3. By the end of the study, 103 unfavorable TB treatment events were recorded: 64 deaths, 35 losses to follow-up, and 4 treatment failures. The team successfully contacted 72 of the 96 cohort members eligible for outcome tracing. Of the 72 people traced, 7 were cured, 13 completed treatment, 2 were reported deceased, and 50 were confirmed to still be on treatment. Treatment outcomes for those who were traced were updated to reflect their outcomes as reported in the tracing survey.

**Table 3:** Distribution of TB treatment outcomes in the full cohort.

Individual TB treatment outcome	n	%
Cured	88	11.4
Treatment completed	228	29.4
Treatment failed	4	0.5
Died	64	8.3
Lost to follow-up	35	4.5
Not evaluated (transferred out or unknown) ^a^	151	19.5
Transferred out ^b^	17	2.2
Treatment stopped due to other medical condition	1	0.1
Still on treatment: Confirmed via tracing survey	50	6.5
Presumed still on treatment: On treatment for less than 6 months and no outcome recorded	122	15.7
No outcome recorded 6 or more months since treatment initiation	15	1.9
**Composite outcome:** Unfavorable TB treatment outcome ^c^	103	13.3

Data are from the 2019 East Africa TB/HIV and Mobility Study.

^a^ Outcome category used in TB treatment registers in Kenya and Tanzania.

^b^ Outcome category used in TB treatment registers in Uganda.

^c^ Composite outcome combines outcomes of treatment failed, died, and lost to follow-up.

**Fig 3:**
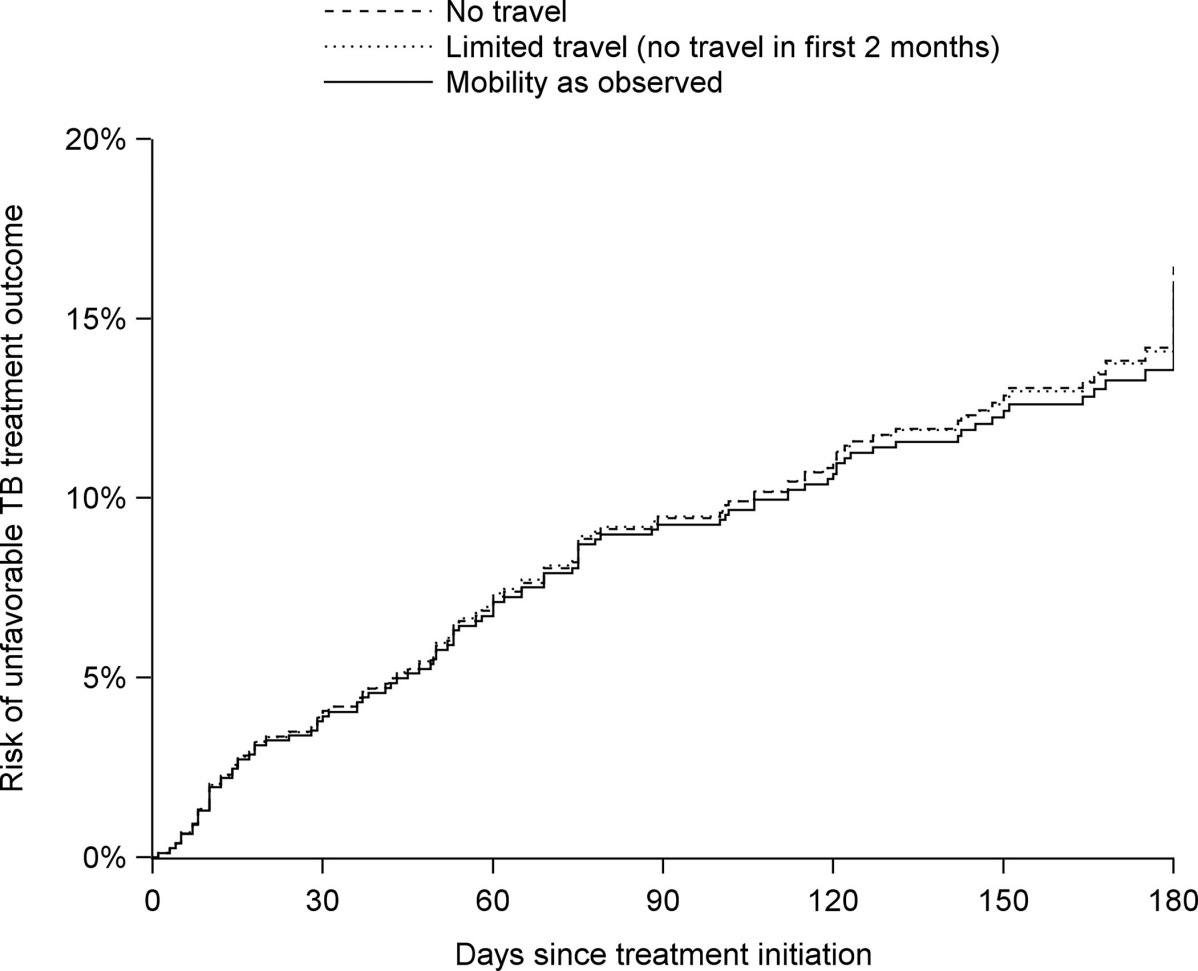
Risk of unfavorable TB treatment outcome following treatment initiation, by mobility pattern. “No travel” indicates no overnight travel outside one’s area of residence in the 6-month period. “Limited travel” indicates no travel in the first 2 months (with travel as observed after the 2-month period). “Mobility as observed” presents the risk under the pattern of mobility observed in the cohort. Data are from the 2019 East Africa TB/HIV and Mobility Study.

Fig 3 presents the risk of unfavorable TB treatment outcome, stratified by mobility pattern, for the 180 days after initiation of TB treatment. Under observed mobility patterns, the risk of an unfavorable TB treatment outcome by 60 days was 7.1% (95% CI: 5.3%, 8.9%), and the risk of an unfavorable TB treatment outcome by 180 days was 15.9% (95% CI: 12.9%, 19.0%) (Table 4). Risks by 60 days were similar under all assessed mobility patterns. At 180 days, risk of unfavorable TB treatment outcomes was slightly higher among those with limited travel (risk difference: 0.5%; 95% CI: -4.1%, 5.1%) and no travel (risk difference: 0.5%; 95% CI: -4.2%, 5.3%).

**Table 4:** Risks of unfavorable TB treatment outcome by 60 and 180 days after treatment initiation.

Mobility pattern in first 6 months following initiation of TB treatment	Risk of unfavorable TB treatment outcome ^a^ (%) (95% CI ^b^)		Risk difference (%) (95% CI ^c^)	
	60-day	180-day	60-day	180-day
Mobility as observed	7.1 (5.3, 8.9)	15.9 (12.9, 19.0)	0	0
Limited travel (no travel in the first 2 months)	7.3 (5.4, 9.3)	16.5 (13.0, 19.9)	0.2 (-2.5, 2.9)	0.5 (-4.1, 5.1)
No travel	7.3 (5.3, 9.2)	16.4 (12.8, 20.1)	0.1 (-2.6, 2.8)	0.5 (-4.2, 5.3)

Data are from the 2019 East Africa TB/HIV and Mobility Study.

^a^ Composite outcome that includes death, loss to follow-up, and treatment failure.

^b^ Standard errors for risks were summarized across 200 imputations using Rubin’s rules.

^c^ Standard errors for risk differences were estimated using the delta method then summarized across 200 imputations using Rubin’s rules.

## Discussion

We used a novel study design to describe prevalence of geographic mobility in a cohort of people on TB treatment in the Lake Victoria region of East Africa and the association between mobility and treatment outcomes. We estimated that 40.7% (95% CI: 33.3%, 49.6%) of people in care for TB traveled overnight outside their area of residence at least once during TB treatment. Travel was more common among fisherfolk and those with extra-pulmonary TB but was not strongly concentrated in specific populations in this region. We also found that in this cohort, lower mobility was not associated with reduced risk of an unfavorable TB treatment outcome.

Across the 6 months described here, we estimated that the proportion of cohort members taking overnight trips outside their area of residence was lowest in the first month following treatment initiation (5.4%; 95% CI: 3.7%, 8.0%). We estimated that only a small proportion of cohort members (2.8%; 95% CI: 1.3%, 6.0%) took a trip longer than 2 weeks within the 2 months following initiation of TB treatment. The first 2 months of treatment coincide with the intensive phase of treatment, which requires more frequent clinical evaluation and drug collection than the later continuation phase. Healthcare workers from the study facilities also reported that they discourage travel among people on TB treatment, especially during the first 2 months of treatment [46].

We estimated that by the sixth month since initiation of TB treatment, the proportion traveling reached almost one-quarter of people in care (23.8%; 95% CI: 16.2%, 34.9%). Several interventions have been proposed to improve TB services for people who travel during treatment. Cell phone applications or other digital technologies could be used [47, 48] to address challenges in treatment adherence related to disruptions of daily routines. Mobile clinics, prolonged clinic hours, and expansion of family-supported treatment or home visits [27] could help to prevent missed visits by increasing opportunities to receive care. Treatment options that reduce the frequency of visits or duration of TB treatment may also be beneficial. Examples include provision of extended drug supplies to people with clinical improvements or smear conversion by 2 months of treatment, or shorter TB regimens, such as the 4-month rifapentine-moxifloxacin regimen recently supported by the World Health Organization [49]. Greater use of electronic medical records [50, 51], more regional coordination of TB treatment cards, and formalization and streamlining of cross-border facility linkages [52] could also simplify facility transfers and drug collection, especially for people who may need to travel unexpectedly. Our results suggest that workers in the fishing industry are more likely than others in TB care to travel overnight outside their area of residence, particularly in the early months of treatment. This population could be considered for a differentiated service delivery approach that is adapted to common travel patterns in this industry. Healthcare workers in the region have indicated that currently, people on TB treatment who expect to travel can request additional medication to cover the duration of travel, and that a client can request a referral letter from their provider in case of extended travel [46, 53]. These accommodations require advanced knowledge of travel plans and notification of one’s provider, however, which may not always be feasible. Current and future strategies for the provision of mobility-sensitive care may benefit from integrating measures of mobility into routine healthcare records or other deliberate data collection efforts.

This study used a two-phase design in which we enumerated a cohort entering TB care and then collected detailed data on exposures and covariates in a subset of that cohort. As a result, those who were not included in the subcohort had missing data by design. We used analytic methods to draw inference from the additional data collected among subcohort members to the broader target population, specifically using covariates collected in both data sources to impute missing data in the full cohort. Under a set of assumptions, namely that conditional on measured covariates, individuals included in the subcohort are exchangeable with those who are not, this approach allows for the generation of results that are not constrained to only the registered TB patients included in the subcohort.

The method we applied to impute missing data is valid provided that, conditional on the variables included in the imputation model, the true values of the imputed variables were independent of the probability that the data were missing. We imputed values for not only the mobility variables that were missing by design, but also for covariates in the model which were not measured or were incompletely measured in the full cohort. This includes variables such as residing near the health facility, which was missing for 35% of cohort members, as well as other covariates measured only in the subcohort survey. We included covariates in our imputation model that we expected to be associated with mobility or missingness of the mobility variable, though it is possible that we did not include all important predictors. The variables collected in the subcohort survey are also subject to social desirability and recall biases common in survey research. In this study, we specifically collected data on overnight trips outside the area of residence. Compared to local travel or travel that did not involve an overnight stay, we presume that overnight, out-of-area trips are more likely to disrupt daily medication routines and interrupt treatment monitoring and drug collection. Our study does not elucidate the mechanism by which mobility may affect TB treatment outcomes. This can be assessed in future studies that examine additional dimensions of mobility and assess the effects of travel intermediate outcomes hypothesized to be along the causal path between travel and TB treatment outcomes, such as the number or frequency of treatment visits or the timing of anti-TB drug collection.

We estimated that 15.9% (95% CI: 12.9%, 19.0%) of cohort members experienced an unfavorable TB treatment outcome by 180 days since initiating TB treatment. In estimating risks under “no travel” and “limited travel” patterns, we found weak, statistically insignificant associations between lower mobility and higher risk of unfavorable treatment outcomes at 60 and 180 days. By 180 days, the risk of an unfavorable TB treatment outcome was 0.5 (95% CI: -4.1, 5.1) percentage points higher under a pattern of limited travel (i.e., no overnight, out-of-area trips for the first 2 months) as compared to the observed mobility pattern. The 180-day risk was also 0.5 (95% CI: -4.2, 5.3) percentage points higher under a pattern of no travel (i.e., no overnight, out-of-area trips for 6 months) as compared to observed mobility. Multiple explanations are possible. Mobility could reduce the risk of unfavorable TB treatment outcomes, if, for example, people are traveling to reach economic opportunities, better health care, or social support. It is also possible that despite reported difficulties in accessing TB care while traveling in the East African region [46, 52, 54, 55], the services offered at the health facilities included in this study were sufficient to mitigate unfavorable treatment outcomes among mobile people in this population. It is also possible that greater mobility is positively associated with unfavorable TB treatment outcomes, and that in this cohort, the generally short duration of travel mitigated the impact of mobility on TB treatment outcomes. The small numbers of long-duration trips and trips during the intensive phase of TB treatment constrain our ability to examine heterogeneity in the mobility-treatment outcome relationship in this cohort. The extent to which the mobility-treatment outcome association varies by different aspects of mobility (e.g., frequency of travel, distance, circular or temporary migration) could be elucidated in future studies that recruit larger numbers of highly mobile people in TB care and measure mobility in greater depth. Furthermore, our results could have been driven by unmeasured factors associated with both limited mobility and unfavorable treatment outcomes, such as baseline health status, wellness during TB treatment, or social or economic factors identified as predictors of unfavorable TB treatment outcomes in other settings [56, 57]. For example, if people in better health or with higher incomes had both a lower risk of unfavorable treatment outcomes and a greater likelihood of traveling, travel would appear to improve outcomes, as the direction of the association we report suggests. We did not specifically measure socioeconomic status in this study; however, we explored two measures that were assessed and may represent some dimensions of socioeconomic status, household hunger and employment status. We found that risk differences across mobility patterns were similar in strata of these variables. Measurement of time-varying disease prognosis and socioeconomic status in future studies would allow for analytic control for these potential confounders and could further elucidate the effect of mobility on treatment outcomes.

We used a composite outcome in this analysis. This composite outcome is operationally useful, given the desirability of reducing all component outcomes (death, loss to follow-up, and treatment failure). It may obscure associations, however, between mobility and individual TB treatment outcomes. We also were not able to definitively ascertain outcomes for all cohort members. Cohort members who were confirmed still on treatment at study closure were censored on the closure date, as were cohort members with no recorded outcome (unfavorable or otherwise) who had not yet reached 6 months on treatment. The outcome categories used in the TB treatment registers also introduced complexity in outcome ascertainment. In Kenya and Tanzania, an outcome of “not evaluated” was recorded for people who transferred out of the facility and for those who had an unknown outcome. In our main analysis, we assumed that cohort members with an outcome of “not evaluated” (n = 151) did not experience an unfavorable TB treatment outcome. We conducted additional analyses to explore the sensitivity of our results to classifying these “not evaluated” outcomes as non-events. The full results of this analysis are shown in S4 Text. Briefly, the mobility-outcome association at 60 days after initiating treatment was similar across all potential misclassification scenarios, while the association at 180 days was sensitive only to differential outcome misclassification and high levels of non-differential misclassification. Across all scenarios, the 60- and 180-day risk differences remained small and the corresponding 95% confidence intervals included the null value.

We applied inverse probability of censoring weights in our analysis. In using this approach, we assumed that unobserved event time (i.e., time after censoring) is exchangeable with observed event time for cohort members with similar baseline characteristics. This assumption relies on correct specification of the censoring model. We explored the sensitivity of our results to specification of the censoring model, repeating the main survival analysis, first, with a restricted set of predictor variables that relies less on imputed covariate values (S3 Fig); and second, with no censoring weights applied (S4 Fig). Results were similar to those presented in the main analysis. It is possible that, in our censoring model, we did not include all variables associated with the outcome and censoring. For example, if lower care-seeking behavior is associated with unfavorable TB treatment outcomes and mobility, and care-seeking is not adequately represented by covariates in the censoring model, our results might underestimate the risk of unfavorable outcome in the “no travel” and “limited travel” arms.

Few studies have assessed mobility among people on TB treatment, and in East Africa, these are limited to qualitative assessments [54, 55]. Our results complement these qualitative findings in that they describe the extent to which people in the Lake Victoria region travel during TB treatment. Furthermore, this study demonstrates an efficient way to overcome limitations in routine data systems and compute generalizable estimates that involve parameters that are not well described in routine data. This and further studies of mobility among people in care for TB may help elucidate the relationship between mobility and TB care experiences and can inform development and implementation of mobility-competent health services in East Africa.

## Supporting information

S1 Text Further details of the subcohort recruitment process. (DOCX)

S2 Text Multiple imputation of monthly mobility indicator variables and covariates. (DOCX)

S3 Text Estimation of inverse probability of censoring weights. (DOCX)

S4 Text Sensitivity analysis re-classifying “not evaluated” outcomes as unfavorable TB treatment outcomes. (DOCX)

S1 Table Trips and nights away per month reported in complete months of mobility data. (DOCX)

S2 Table Cohort members traveling in each month since TB treatment initiation. (DOCX)

S1 Fig Trips during TB treatment fully or partly attributed to various reasons. Results are presented for 80 of 81 reported trips (no reason was reported for 1 trip). Data are from the 2019 East Africa TB/HIV and Mobility Study.
(TIFF)

S2 Fig Percentages of cohort members initiating a trip in each month since TB treatment initiation, by characteristics. Results are disaggregated by a) sex; b) HIV status; c) TB site; d) recent participation in the fishing industry; and e) whether residing in the same subcounty/district as the health facility where TB treatment was initiated. Data are from the 2019 East Africa TB/HIV and Mobility Study.
(TIF)

S3 Fig Risk of unfavorable TB treatment outcome by mobility pattern, with only register variables used to compute censoring weights. To assess the sensitivity of results to the inclusion of imputed covariate values when computing censoring weights, we repeated the survival analysis, limiting the predictor variables in the censoring model to variables widely available from TB treatment registers. The censoring model used to produce this figure included the covariates country sex, age, HIV status, and pulmonary vs. extra-pulmonary TB. Data are from the 2019 East Africa TB/HIV and Mobility Study.
(TIF)

S4 Fig Risk of unfavorable TB treatment outcome by mobility pattern, with no censoring weights applied. To assess the sensitivity of results to the censoring weights applied, we repeated the survival analysis while excluding these weights. This figure shows the risks when no censoring weights are applied in the analysis. Data are from the 2019 East Africa TB/HIV and Mobility Study.
(TIF)
