# Supplementary material for: Geographic mobility and treatment outcomes among people in care for tuberculosis in the Lake Victoria region of East Africa: A multi-site prospective cohort study
Source: PLOS Glob Public Health. 2023 Jun 5;3(6):e0001992. doi: 10.1371/journal.pgph.0001992 (PMC10241360; doi:10.1371/journal.pgph.0001992)
Supplement: S1 Text — (DOCX) [file pgph.0001992.s001.docx]

# S1 Text. Further details of the subcohort recruitment process

Resource constraints precluded us from interviewing all cohort members. Given an intended total subcohort size of 300 people, we set survey recruitment targets for each health facility proportionate to the number of people on treatment. Each health facility target was halved to produce target numbers of cohort members with and without HIV to be recruited for the subcohort survey.

All survey recruitment procedures were performed at the health facilities. As people on TB treatment visited a study facility for routine care, health facility staff consulted TB treatment records to identify whether the person met the eligibility criteria for the study cohort. People identified as cohort members were referred to a member of the study team, who then confirmed cohort membership and administered consent and recruitment procedures. Recruitment continued at each facility until the targets were achieved. The survey participants constituted a subcohort (n = 301) within the full analytic cohort (n = 775).
