# Supplementary material for: Geographic mobility and treatment outcomes among people in care for tuberculosis in the Lake Victoria region of East Africa: A multi-site prospective cohort study
Source: PLOS Glob Public Health. 2023 Jun 5;3(6):e0001992. doi: 10.1371/journal.pgph.0001992 (PMC10241360; doi:10.1371/journal.pgph.0001992)
Supplement: S2 Text — (DOCX) [file pgph.0001992.s002.docx]

# S2 Text. Multiple imputation of monthly mobility indicator variables

We performed multiple imputation by fully conditional specification (FCS) to impute missing values for covariates in the imputation model with incomplete data and for mobility indicator variables. The mobility indicator variables for the main analysis indicated whether the cohort member initiated any overnight trips outside the area of residence in the first, second, third, fourth, fifth, and sixth 30-day period (approximate “months”) following the date on which they initiated TB treatment.

Table A illustrates the data structure for the imputation. For each subcohort member, mobility data were imputed for person-months that were only partially reported on or which occurred after their participation in the survey. For cohort members who did not participate in the survey, values for all 6 person-month mobility variables were imputed. Imputation models for the mobility variables included a combination of covariates assessed in the full analytic cohort and in the subcohort. Following the imputation procedure for the main mobility variables, we repeated the process to impute values for two additional sets of mobility variables which indicated, for each month 1 through 6, whether someone had spent more than 7 total nights or more than 14 consecutive nights away from their residential area.

**Table A.** **Illustration of data structure indicating where missing values were imputed.**

|  | | **Covariates with complete data** | **Covariates with missing data** | Mobility indicator variable ^a^ for month ^b^: | | | | | |
| --- | --- | --- | --- | --- | --- | --- | --- | --- | --- |
|  |  |  |  | **1** | **2** | **3** | **4** | **5** | **6** |
| Subcohort members  (n = 301) | Cohort member 1 | Z | Y | 0 | 0 | 0 | 0 | 0 | 1 |
|  | Cohort member 2 | Z | i ^c^ | 1 | 0 | 0 | 0 | i | i |
|  | … |  |  | … | … | … | … | … | … |
|  | Cohort member 300 | Z | Y | 0 | 1 | 0 | 0 | 1 | i |
|  | Cohort member 301 | Z | Y | i | 0 | 1 | 0 | 0 | 0 |
| Cohort members who did not participate in the survey  (n = 474) | Cohort member 302 | Z | i | i | i | i | i | i | i |
|  | Cohort member 304 | Z | i | i | i | i | i | i | i |
|  | … |  |  | … | … | … | … | … | … |
|  | Cohort member 774 | Z | i | i | i | i | i | i | i |
|  | Cohort member 775 | Z | i | i | i | i | i | i | i |

^a^ Coded as 0 or 1 to indicate whether the cohort member took an overnight trip outside their area of residence in that month.

^b^ Month since TB treatment initiation.

^c^ Impute.

Table B summarizes the variables used in the multivariate imputation model and for each incomplete variable, the variable-specific imputation method used. Models for each incomplete variable included all other variables in the table. We created 200 “complete” imputed data sets, allowing uncertainty in the imputation process to propagate through to final confidence intervals around our estimates.

**Table B. Coding and methods for multiple imputation of missing mobility and covariate data.**

| **Variable** | **Type** | **Coding for imputation models** | **Missing values?** | **Method for imputation** |
| --- | --- | --- | --- | --- |
| Sex | Binary | Female versus Male | No | N/A^a^ |
| Age | Continuous | Linear predictor and 3 restricted quadratic spline basis functions | No | N/A |
| Bacteriologic confirmation of TB | Binary | Whether TB diagnosis was noted as bacteriologically confirmed (by GeneXpert, culture, smear, or unspecified method) | No | N/A |
| HIV status | Binary | HIV-positive versus HIV-negative | No | N/A |
| Country where initiating TB treatment | Nominal | Kenya, Tanzania, or Uganda | No | N/A |
| Outcome indicator variable | Nominal | Experienced an unfavorable TB treatment outcome, experienced a competing event, or was censored at time *t* | No | N/A |
| Cumulative baseline hazard of unfavorable TB treatment outcome | Continuous | Approximated by the Nelson–Aalen estimator of the hazard at time | No | N/A |
| Resided near health facility | Binary | Resided in the same district (if in Uganda or Tanzania) or subcounty (if in Kenya) as the health facility where enrolled in TB treatment versus Resided further away | Yes | Discriminant function |
| Partnered | Binary | Married or cohabitating with a sexual partner versus Single, divorced, separated, or widowed | Yes | Discriminant function |
| Employment status | Nominal | Formally employed, informally employed, not employed and seeking work, or not employed and not seeking work | Yes | Discriminant function |
| Recent work in the fishing industry | Binary | Worked in the fishing industry in the past 12 months, versus Did not work in this industry in the past 12 months (including those who did not work at all in the past 12 months) | Yes | Discriminant function |
| Household hunger | Binary | Any member of the household went to bed hungry in the past 30 days versus No household members went to bed hungry in the past 30 days | Yes | Discriminant function |
| Recently remained home | Binary | Remained home all day for the most of the last 7 days versus Leaving home for part of most days | Yes | Discriminant function |
| Recent night away from home | Binary | Slept away from home (irrespective of location/proximity to cohort member’s residential area) in the past 30 days versus Only slept at home in the past 30 days | Yes | Discriminant function |
| Clinic presentation delay | Binary | Visited a health facility for care within 6 weeks of TB symptom onset, versus Presented to a health facility later | Yes | Discriminant function |
| TB medication adherence (6 variables, one for each month since treatment initiation) | Binary | Perfect adherence during the month, versus Imperfect adherence during the month (self-reported) | Yes | Discriminant function |
| Educational attainment | Ordinal | Less than primary school; Primary school; Form 6; or College, vocational or tertiary school | Yes | Logistic |
| TB site | Binary | Pulmonary versus Extrapulmonary | Yes | Discriminant function |
| Patient type | Binary | New versus Relapse or return after treatment failure or loss to follow-up | Yes | Discriminant function |
| Mobility indicator variable for any travel during the month (6 variables, one for each month since treatment initiation) | Binary | Overnight travel outside one’s residential area during the month, versus No overnight travel outside one’s residential area during the month (self-reported) | Yes | Discriminant function |
| Additional mobility indicator variable for descriptive analysis: Total of >7 nights away (6 variables, one for each month since treatment initiation) | Binary | Overnight travel outside one’s residential area for a total of more than 7 nights during the month, versus Travel for 0 to 7 nights only (self-reported) | Yes | Discriminant function |
| Additional mobility indicator variable for descriptive analysis: >14 consecutive nights away (2 variables, for months 1 and 2 since treatment initiation) | Binary | Overnight travel outside one’s residential area for a total of more than 14 consecutive days during the month, versus Travel for 0 to 14 consecutive nights only (self-reported) | Yes | Discriminant function |

^a^ Not applicable; no missing values to impute.

To assess the quality of the imputations, we examined the range of imputed values for each variable to confirm plausibility, and then we assessed whether any variables had large differences between the means and variances of the observed and imputed values [1]. For each binary variable with imputed values, we calculated the mean (i.e., the proportion of observations in one of the two levels) and standard deviation of the observed values and, in each imputation, the mean and standard deviation of the imputed values. We assessed, for each imputed variable and imputation, whether the absolute difference in means between the observed and imputed values was within 2 standard deviations of the original mean [2]. The means were within 2 standard deviations for all binary variables used in the imputation model.

**References:**

1. Liu Y, De A. Multiple Imputation by Fully Conditional Specification for Dealing with Missing Data in a Large Epidemiologic Study. Int J Stat Med Res. **2015**; 4(3):287–295.

2. Stuart EA, Azur M, Frangakis C, Leaf P. Multiple imputation with large data sets: A case study of the children’s mental health initiative. Am J Epidemiol. **2009**; 169(9):1133–1139.
