# Supplementary material for: Geographic mobility and treatment outcomes among people in care for tuberculosis in the Lake Victoria region of East Africa: A multi-site prospective cohort study
Source: PLOS Glob Public Health. 2023 Jun 5;3(6):e0001992. doi: 10.1371/journal.pgph.0001992 (PMC10241360; doi:10.1371/journal.pgph.0001992)
Supplement: S3 Text — (DOCX) [file pgph.0001992.s003.docx]

# S3 Text. Estimation of inverse probability of censoring weights

To examine risks under each mobility patterns of interest, we assessed the distribution of unfavorable TB treatment outcomes that occurred during periods of follow-up that were consistent with the mobility pattern. We discretized follow-up into periods of 30 days to operationalize the artificial censoring. Whenever the mobility data indicated a deviation from the mobility pattern defining a given arm, we artificially censored the record at the end of the month (i.e., 30-day period) in which the deviation occurred. For example, in the “no travel in the first 2 months” arm, we included:

- Up to one month of follow-up for any person who traveled in their first month following treatment initiation (by day 30)*;
- Up to two months of follow-up* for any person who first traveled in their second month following treatment initiation (i.e., between days 31 and 60);
- All follow-up time for people who did not travel; and
- All follow-up time for people who first traveled in the third, fourth, fifth or sixth month following treatment initiation.

*Their observed follow-up time was used if they experienced their first unfavorable treatment outcome or non-artificial censoring event (transfer out, outcome listed as not evaluated or unknown, 200 days beyond treatment initiation, or study closure) within the month.

This artificial censoring may lead us to underestimate the risk of unfavorable TB treatment outcomes in mobility pattern. We used inverse probability of censoring weights to estimate the risk of unfavorable TB treatment outcomes that would have been observed under each mobility pattern absent the artificial censoring mechanism. We fit a pooled logit model for censoring, using as model covariates, hypothesized predictors of the artificial censoring mechanism (i.e., travel) and the outcome. Specifically, we included the sex, age, HIV status, educational attainment, household hunger, employment status, recent work in the fishing industry, and clinic presentation delay at the emergence of TB symptoms. We estimated the inverse probability of censoring weights as the probability of censoring at the end of the discretized time period divided by the conditional probability of censoring at that time, given values of the covariates in the censoring model. Under the assumption of correct model specification, the weighted data represent the distribution of outcomes that would have been observed for each mobility pattern, absent the artificial censoring.
