# Supplementary material for: Geographic mobility and treatment outcomes among people in care for tuberculosis in the Lake Victoria region of East Africa: A multi-site prospective cohort study
Source: PLOS Glob Public Health. 2023 Jun 5;3(6):e0001992. doi: 10.1371/journal.pgph.0001992 (PMC10241360; doi:10.1371/journal.pgph.0001992)
Supplement: S4 Text — (DOCX) [file pgph.0001992.s004.docx]

**S4 Text.** **Sensitivity analysis re-classifying “not evaluated” outcomes as** **unfavorable TB treatment outcomes.**

The Table below shows the results of a quantitative bias analysis assessing the impact of potential misclassification of unfavorable treatment outcomes as non-events among people with a recorded TB treatment outcome of “not evaluated.” We re-estimated the 60- and 180-day risks and risk differences across a range of probabilities that people with recorded outcomes of “not evaluated” experienced unfavorable TB treatment outcomes. We explored non-differential misclassification scenarios as well as scenarios in which the probability of misclassification differed between mobile and non-mobile people. When reclassifying outcomes, we assumed that any unfavorable treatment outcome occurred at the end of follow-up (i.e., at the censoring date used in the main analysis).

**Table.****Risks of an unfavorable TB treatment outcome under outcome misclassification scenarios.**

| **Scenario** | **Probability of**  **misclassification ^a^** | | **Mobility pattern in first 6 months following initiation of TB treatment** | **Risk of unfavorable TB treatment outcome ^b^ (%) (95% CI ^c^)** | | **Risk difference (%) (95% CI ^d^)** | |
| --- | --- | --- | --- | --- | --- | --- | --- |
|  | **Among mobile cohort members** | **Among non-mobile cohort members** |  | **60-day** | **180-day** | **60-day** | **180-day** |
| Ref ^e^ | 0 | 0 | Mobility as observed | 7.1 (5.3, 8.9) | 15.9 (12.9, 19.0) | 0 | 0 |
|  |  |  | Limited travel (no travel in the first 2 months) | 7.3 (5.4, 9.3) | 16.5 (13.0, 19.9) | 0.2 (-2.5, 2.9) | 0.5 (-4.1, 5.1) |
|  |  |  | No travel | 7.3 (5.3, 9.2) | 16.4 (12.8, 20.1) | 0.1 (-2.6, 2.8) | 0.5 (-4.2, 5.3) |
| A | 0.25 | 0.25 | Mobility as observed | 7.5 (5.6, 9.4) | 24.4 (19.8, 29.1) | 0 | 0 |
|  |  |  | Limited travel (no travel in the first 2 months) | 7.7 (5.7, 9.8) | 24.9 (19.9, 29.9) | 0.2 (-2.6, 3.0) | 0.4 (-5.4, 6.2) |
|  |  |  | No travel | 7.7 (5.7, 9.7) | 24.7 (19.5, 29.9) | 0.1 (-2.6, 2.8) | 0.2 (-5.8, 6.3) |
| B | 0.5 | 0.5 | Mobility as observed | 8.0 (6.0, 10.0) | 32.1 (27.1, 37.1) | 0 | 0 |
|  |  |  | Limited travel (no travel in the first 2 months) | 8.2 (6.0, 10.3) | 32.4 (27.1, 37.8) | 0.2 (-2.7, 3.0) | 0.3 (-6.0, 6.7) |
|  |  |  | No travel | 8.1 (6.0, 10.1) | 32.1 (26.4, 37.8) | 0.1 (-2.7, 2.9) | 0.0 (-6.6, 6.7) |
| C | 0.75 | 0.75 | Mobility as observed | 8.4 (6.4, 10.4) | 39.1 (34.3, 43.9) | 0 | 0 |
|  |  |  | Limited travel (no travel in the first 2 months) | 8.6 (6.4, 10.7) | 39.3 (34.1, 44.5) | 0.1 (-2.7, 3.0) | 0.2 (-6.4, 6.8) |
|  |  |  | No travel | 8.5 (6.4, 10.6) | 38.8 (33.2, 44.5) | 0.1 (-2.7, 2.9) | -0.2 (-7.1, 6.7) |
| D | 1 | 1 | Mobility as observed | 8.8 (6.8, 10.8) | 45.3 (40.8, 49.8) | 0 | 0 |
|  |  |  | Limited travel (no travel in the first 2 months) | 9.0 (6.8, 11.1) | 45.4 (40.5, 50.3) | 0.1 (-2.8, 3.1) | 0.1 (-6.5, 6.7) |
|  |  |  | No travel | 8.9 (6.8, 11.0) | 44.9 (39.5, 50.2) | 0.1 (-2.8, 3.0) | -0.4 (-7.4, 6.5) |
| E | 0.25 | 0 | Mobility as observed | 7.4 (5.5, 9.3) | 19.8 (15.9, 23.8) | 0 | 0 |
|  |  |  | Limited travel (no travel in the first 2 months) | 7.6 (5.6, 9.6) | 20.0 (15.7, 24.3) | 0.2 (-2.5, 2.9) | 0.1 (-5.1, 5.4) |
|  |  |  | No travel | 7.5 (5.6, 9.5) | 19.2 (14.8, 23.6) | 0.1 (-2.6, 2.8) | -0.6 (-6.1, 4.8) |
| F | 0.5 | 0 | Mobility as observed | 7.7 (5.8, 9.6) | 23.5 (19.0, 28.0) | 0 | 0 |
|  |  |  | Limited travel (no travel in the first 2 months) | 7.9 (5.8, 10.0) | 23.3 (18.5, 28.1) | 0.2 (-2.6, 3.0) | -0.2 (-5.9, 5.5) |
|  |  |  | No travel | 7.8 (5.8, 9.8) | 21.8 (16.8, 26.8) | 0.1 (-2.6, 2.8) | -1.7 (-7.7, 4.3) |
| G | 0.75 | 0 | Mobility as observed | 8.0 (6.0, 10.0) | 27.1 (22.1, 32.0) | 0 | 0 |
|  |  |  | Limited travel (no travel in the first 2 months) | 8.1 (6.0, 10.2) | 26.5 (21.2, 31.7) | 0.2 (-2.7, 3.0) | -0.6 (-6.6, 5.4) |
|  |  |  | No travel | 8.1 (6.0, 10.1) | 24.3 (18.8, 29.8) | 0.1 (-2.7, 2.9) | -2.8 (-9.2, 3.6) |
| H | 1 | 0 | Mobility as observed | 8.3 (6.3, 10.3) | 30.5 (25.1, 35.8) | 0 | 0 |
|  |  |  | Limited travel (no travel in the first 2 months) | 8.4 (6.2, 10.5) | 29.5 (23.8, 35.3) | 0.1 (-2.7, 3.0) | -0.9 (-7.2, 5.4) |
|  |  |  | No travel | 8.3 (6.2, 10.4) | 26.8 (20.6, 32.9) | 0.1 (-2.8, 2.9) | -3.7 (-11, 3.2) |
| I | 0 | 0.25 | Mobility as observed | 7.2 (5.4, 9.1) | 20.8 (16.6, 25.0) | 0 | 0 |
|  |  |  | Limited travel (no travel in the first 2 months) | 7.5 (5.5, 9.5) | 21.6 (16.9, 26.2) | 0.2 (-2.5, 3.0) | 0.8 (-4.6, 6.2) |
|  |  |  | No travel | 7.4 (5.4, 9.3) | 22.1 (17.0, 27.2) | 0.1 (-2.5, 2.8) | 1.3 (-4.3, 7.0) |
| J | 0 | 0.5 | Mobility as observed | 7.4 (5.5, 9.3) | 25.4 (20.5, 30.3) | 0 | 0 |
|  |  |  | Limited travel (no travel in the first 2 months) | 7.6 (5.6, 9.7) | 26.4 (21.0, 31.8) | 0.2 (-2.5, 3.0) | 1.0 (-4.9, 6.9) |
|  |  |  | No travel | 7.5 (5.6, 9.5) | 27.4 (21.6, 33.3) | 0.1 (-2.5, 2.8) | 2.1 (-4.1, 8.3) |
| K | 0 | 0.75 | Mobility as observed | 7.5 (5.6, 9.5) | 29.8 (24.5, 35.0) | 0 | 0 |
|  |  |  | Limited travel (no travel in the first 2 months) | 7.8 (5.7, 9.8) | 31.0 (25.3, 36.8) | 0.2 (-2.5, 3.0) | 1.2 (-5.0, 7.5) |
|  |  |  | No travel | 7.7 (5.7, 9.7) | 32.5 (26.4, 38.6) | 0.1 (-2.6, 2.9) | 2.7 (-3.9, 9.3) |
| L | 0 | 1 | Mobility as observed | 7.7 (5.7, 9.6) | 33.8 (28.4, 39.3) | 0 | 0 |
|  |  |  | Limited travel (no travel in the first 2 months) | 7.9 (5.8, 10.0) | 35.2 (29.3, 41.1) | 0.2 (-2.6, 3.0) | 1.4 (-5.1, 7.9) |
|  |  |  | No travel | 7.8 (5.8, 9.9) | 37.0 (30.9, 43.2) | 0.2 (-2.6, 2.9) | 3.2 (-3.6, 10.0) |

Data are from the 2019 East Africa TB/HIV and Mobility Study.

^a^ Probability that unfavorable TB treatment outcomes were misclassified as non-events.

^b^ Composite outcome that includes death, loss to follow-up, and treatment failure.

^c^ Standard errors for risks were summarized across 200 imputations using Rubin’s rules.

^d^ Standard errors for risk differences were estimated using the delta method then summarized across 200 imputations using Rubin’s rules.

^e^ For the purpose of comparison, this row shows the results presented in the main analysis.

Under increasing probabilities of misclassification of events as non-events, the risk of an unfavorable treatment outcome increased, as would be expected. In the most extreme case (Scenario D), where all events classified as “not evaluated” were, in truth, unfavorable TB treatment outcomes, the 180-day risk of an unfavorable outcome under the observed mobility pattern in the cohort would have been 45.3% (95% CI: 40.8%, 49.8%), as compared to 15.9% (95% CI: 12.9%, 19.0%) as presented in our main analysis (Scenario Ref).

The estimated 60-day risk differences remained within 0.1 percentage point of the results presented in the main analysis under all scenarios. The 180-day risk differences varied to a greater extent. Under high probabilities of non-differential misclassification (Scenarios C and D), the direction of the association comparing the 180-day risks under observed mobility versus no travel was reversed relative to the results presented in the main analysis. Under scenarios where unfavorable outcomes were more commonly misclassified as non-events among people who traveled (Scenarios E, F, G, and H), the direction of the 180-day risk difference for one or both mobility pattern comparisons also reversed. Under scenarios where misclassification was more likely among cohort members who did not travel (Scenarios I, J, K, and L), the direction of the 180-day risk differences remained the same as reported in the main analysis, while the magnitude of the risk differences increased. In all scenarios, the 60- and 180-day risk differences remained small (less than 4 percentage points), and the corresponding 95% confidence intervals included the null value.
