# Supplementary material for: Geographic mobility and treatment outcomes among people in care for tuberculosis in the Lake Victoria region of East Africa: A multi-site prospective cohort study
Source: PLOS Glob Public Health. 2023 Jun 5;3(6):e0001992. doi: 10.1371/journal.pgph.0001992 (PMC10241360; doi:10.1371/journal.pgph.0001992)
Supplement: S1 Table — (DOCX) [file pgph.0001992.s005.docx]

# **S1** Table. Trips and nights away per month reported in complete months of mobility data.

|  | Male subcohort members | Female subcohort members | Subcohort members with HIV-associated TB | Subcohort members without HIV | All subcohort members |
| --- | --- | --- | --- | --- | --- |
| Person-months with mobility data | 565 | 415 | 509 | 471 | 980 |
| Mean (SD ^a^) person-months of mobility data | 3.2 (1.6) | 3.3 (1.6) | 3.2 (1.6) | 3.3 (1.6) | 3.3 (1.6) |
| Mean (SD) number of trips ^b^ initiated per month across all person-months ^c^ | 0.1 (0.4) | 0.1 (0.2) | 0.1 (.3) | 0.1 (0.4) | 0.1 (0.3) |
| Percent (N) of person-months in which a trip was initiated | 8.5% (48) | 5.5% (23) | 6.4% (33) | 8.1% (38) | 7.2% (71) |
| Mean (SD) number of trips initiated per month, in months in which a trip was initiated | 1.2 (0.6) | 1.0 (0) | 1.1 (0.2) | 1.2 (0.7) | 1.1 (0.5) |
| Mean (SD) number of nights away per person-month ^d^ | 0.9 (4.0) | 0.5 (3.0) | 0.6 (3.0) | 0.9 (4.1) | 0.8 (3.6) |
| Percent (N) of person-months in which any nights were spent away ^e^ | 10.3% (58) | 7.2% (30) | 7.9% (40) | 10.2% (48) | 9.0% (88) |
| Mean (SD) number of nights away per month, in months in which any nights were spent away | 9.0 (9.1) | 7.5 (8.7) | 7.6 (8.1) | 9.3 (9.6) | 8.5 (8.9) |

Data are from n = 301 subcohort members in the 2019 East Africa TB/HIV and Mobility Study. These results do not incorporate any imputed values.

^a^ Standard deviation

^b^ Overnight travel outside the subcohort member’s geographic area of residence (district if residing in Tanzania or Uganda, sub-county if residing in Kenya).

^c^ Mean includes values of 0 trips for person-months in which no trips were initiated.

^d^ Mean includes values of 0 nights away for person-months in which no nights were spent away.

^e^ Nights away could span multiple months for a trip initiated in one month but concluding in a different month.
