# Supplementary material for: Geographic mobility and treatment outcomes among people in care for tuberculosis in the Lake Victoria region of East Africa: A multi-site prospective cohort study
Source: PLOS Glob Public Health. 2023 Jun 5;3(6):e0001992. doi: 10.1371/journal.pgph.0001992 (PMC10241360; doi:10.1371/journal.pgph.0001992)
Supplement: S2 Table — (DOCX) [file pgph.0001992.s006.docx]

# S2 Table. Cohort members traveling in each month since TB treatment initiation.

| Month after initiation of TB treatment | Cohort members initiating a trip | | Cohort members initiating a trip of more than 14 consecutive nights | | Cohort members traveling for more than 7 total nights in this month (%) (95% CI) |
| --- | --- | --- | --- | --- | --- |
|  | in this month (%) (95% CI) | in or before this month (%) (95% CI) | in this month (%) (95% CI) | in or before this month (%) (95% CI) |  |
| 1 | 5.4 (3.7, 8.0) | 5.4 (3.7, 8.0) | 1.3 (0.4, 4.1) | 1.3 (0.4, 4.1) | 1.5 (0.5, 4.3) |
| 2 | 8.0 (5.2, 12.3) | 10.1 (7.2, 14.3) | 1.3 (0.4, 4.2) | 2.8 (1.3, 6.0) | 2.9 (1.4, 6.0) |
| 3 | 7.7 (4.8, 12.4) | 13.9 (10.3, 18.9) |  |  | 3.2 (1.6, 6.5) |
| 4 | 14.3 (9.4, 21.7) | 20.8 (15.7, 27.5) |  |  | 2.3 (1.1, 4.9) |
| 5 | 9.7 (6.5, 14.7) | 22.7 (17.4, 29.6) |  |  | 3.3 (1.7, 6.7) |
| 6 | 23.8 (16.2, 34.9) | 40.7 (33.3, 49.6) |  |  | 15.6 (11.1, 22.1) |

“Trips” and “travel” are defined as overnight travel outside one’s sub-county/district of residence. Data are from the 2019 East Africa TB/HIV and Mobility Study.
