# Supplementary figures and images for: Geographic mobility and treatment outcomes among people in care for tuberculosis in the Lake Victoria region of East Africa: A multi-site prospective cohort study
Source: PLOS Glob Public Health. 2023 Jun 5;3(6):e0001992. doi: 10.1371/journal.pgph.0001992 (PMC10241360; doi:10.1371/journal.pgph.0001992)

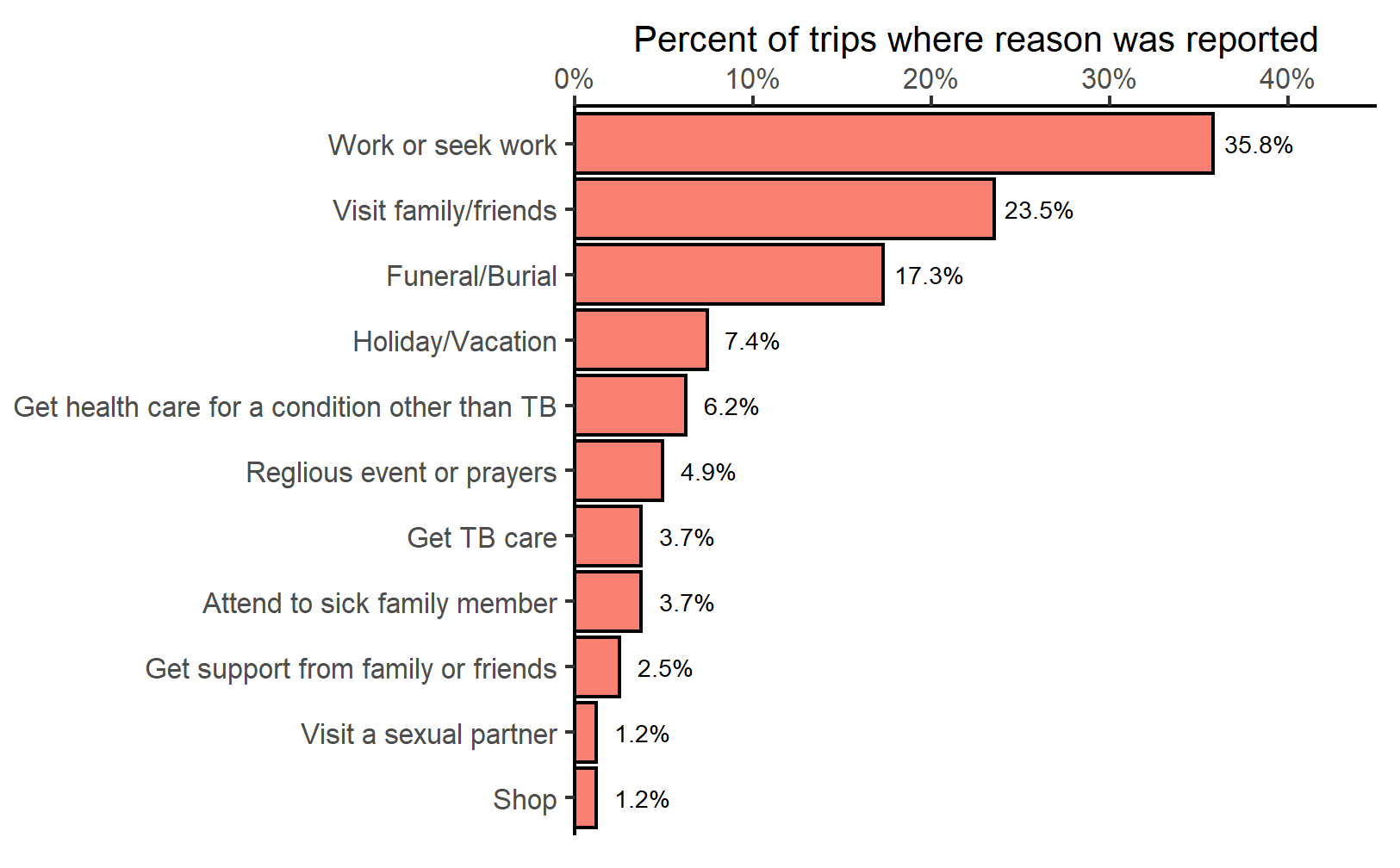

Supplement: S1 Fig — Results are presented for 80 of 81 reported trips (no reason was reported for 1 trip). Data are from the 2019 East Africa TB/HIV and Mobility Study. (TIFF) [file pgph.0001992.s007.tiff]

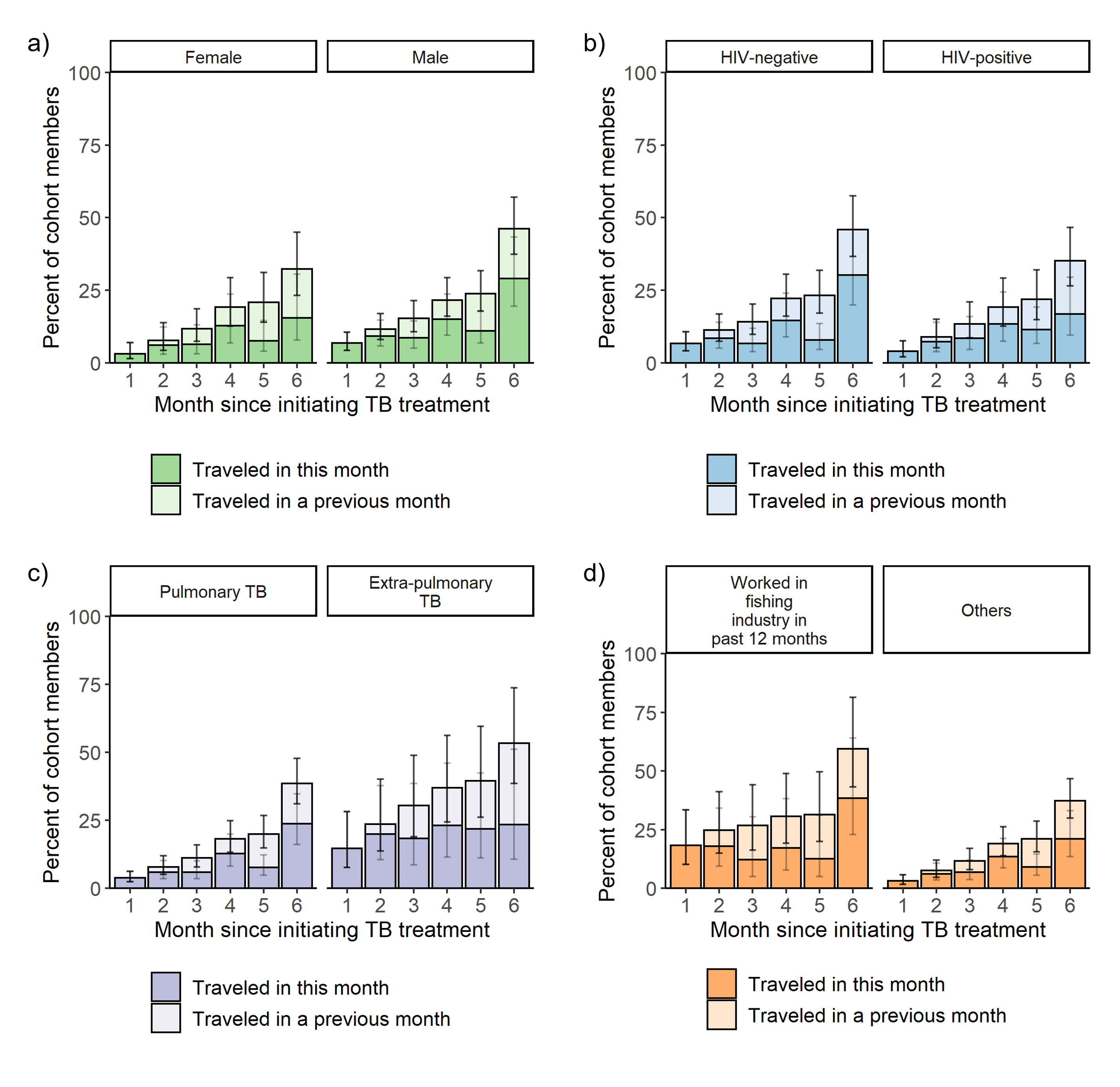

Supplement: S2 Fig — Results are disaggregated by a) sex; b) HIV status; c) TB site; d) recent participation in the fishing industry; and e) whether residing in the same subcounty/district as the health facility where TB treatment was initiated. Data are from the 2019 East Africa TB/HIV and Mobility Study. (TIF) [file pgph.0001992.s008.tif]

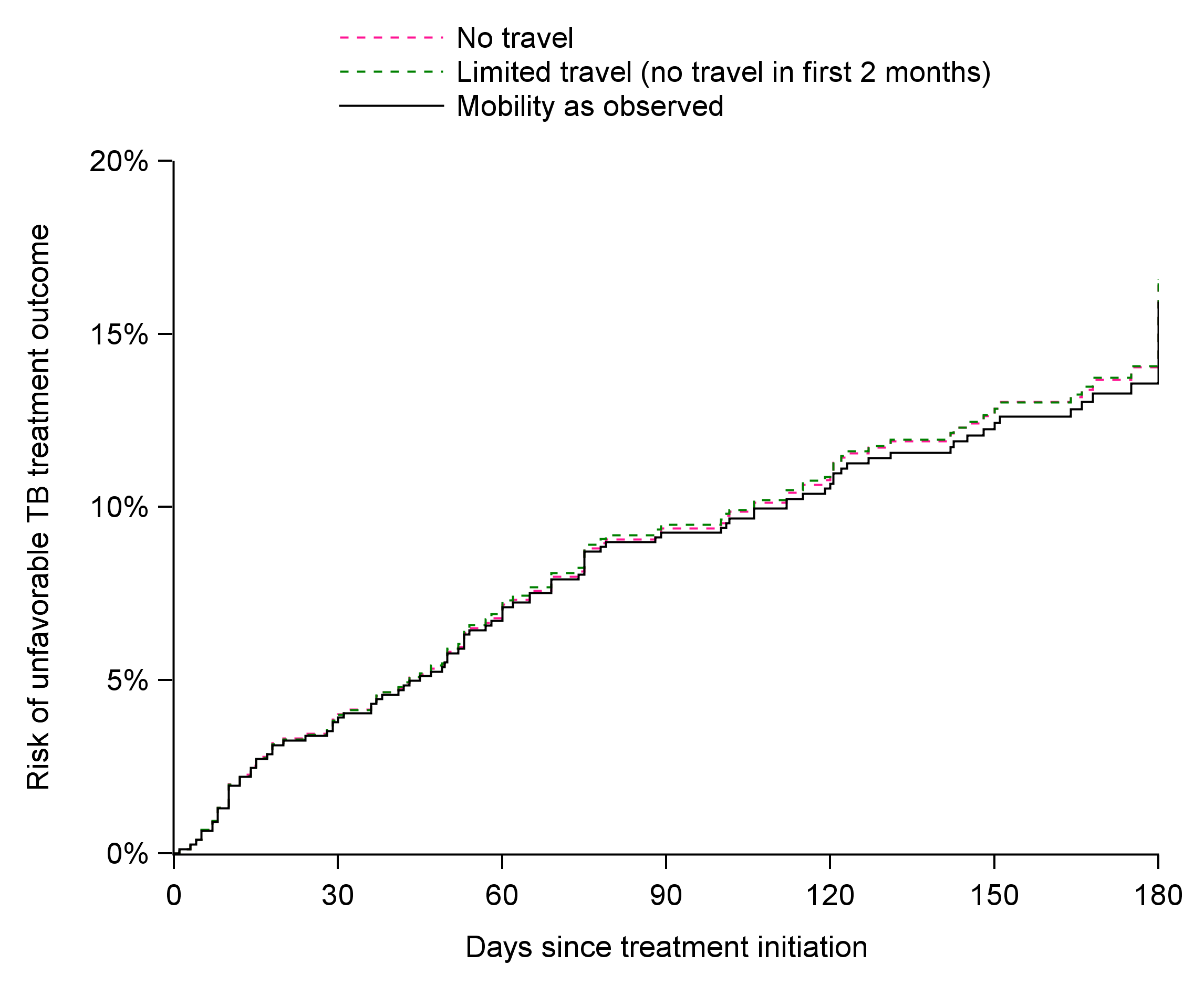

Supplement: S3 Fig — To assess the sensitivity of results to the inclusion of imputed covariate values when computing censoring weights, we repeated the survival analysis, limiting the predictor variables in the censoring model to variables widely available from TB treatment registers. The censoring model used to produce this figure included the covariates country sex, age, HIV status, and pulmonary vs. extra-pulmonary TB. Data are from the 2019 East Africa TB/HIV and Mobility Study. (TIF) [file pgph.0001992.s009.tif]

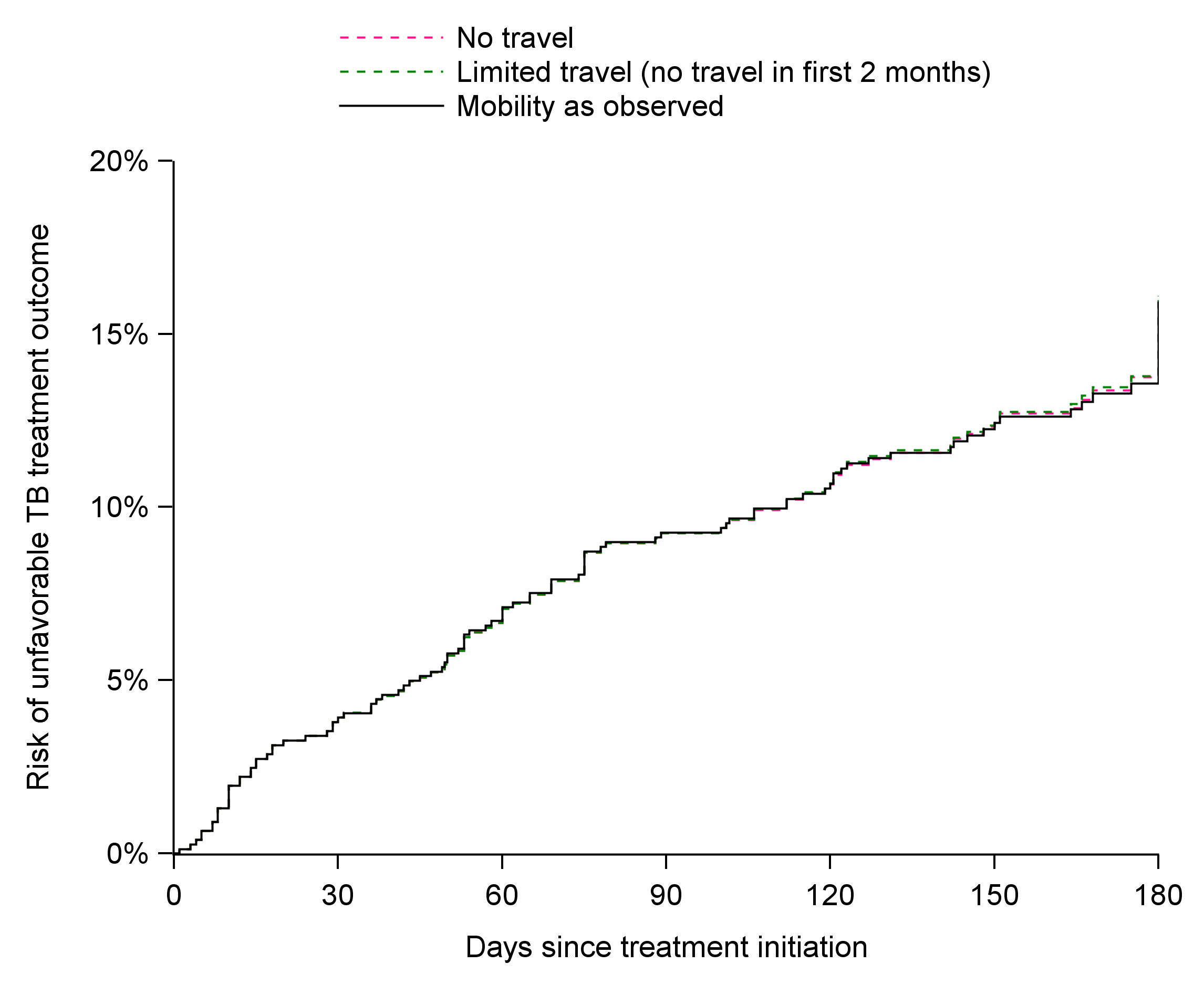

Supplement: S4 Fig — To assess the sensitivity of results to the censoring weights applied, we repeated the survival analysis while excluding these weights. This figure shows the risks when no censoring weights are applied in the analysis. Data are from the 2019 East Africa TB/HIV and Mobility Study. (TIF) [file pgph.0001992.s010.tif]
